# Disseminated *Mycobacterium genavense* infection in a guinea pig (*Cavia porcellus*): a case report

**DOI:** 10.1186/s12917-022-03198-4

**Published:** 2022-03-10

**Authors:** David J. Minich, Alea Agrawal, Stephen A. Kania, Adrien-Maxence Hespel, Andrew Cushing, Dory Meraz, Julie Sheldon

**Affiliations:** 1 Department of Small Animal Clinical Sciences, University of Tennessee College of Veterinary Medicine, 2407 River Drive, TN 37996 Knoxville, USA; 2 Department of Biomedical and Diagnostic Sciences, University of Tennessee College of Veterinary Medicine, 2407 River Dr, Knoxville, TN 37996 USA

**Keywords:** *Cavia porcellus*, Guinea pig, Mycobacteria, *Mycobacterium genavense*

## Abstract

**Background:**

Mycobacteria are found in many environmental conditions and infect a variety of species, including rodents and rabbits. Guinea pigs are used experimentally as a model for *Mycobacterium tuberculosis*, but natural mycobacteriosis in guinea pigs has not been reported.

**Case presentation:**

A 1.5-year-old female guinea pig was found acutely deceased with no premonitory illness. On gross post-mortem examination, multifocal to coalescing, raised, firm, pale tan nodules with discrete, irregular margins were noted over the surfaces of all lung lobes. Histopathology revealed nodules composed of clustered foamy macrophages and multinucleated giant cells containing numerous bacterial rods. Similar bacteria-laden macrophages were noted within sections of the liver, heart, palpebral conjunctiva, duodenum, and cecum. Polymerase chain reaction was performed on tissues collected during post-mortem examination. The 16S rRNA gene product was sequenced and was identical to the *Mycobacterium genavense* type strain.

**Conclusions:**

To the best of the author’s knowledge, this report details the first documented case of *Mycobacterium genvaense* infection in a guinea pig and a follow up investigation of close-contact animals. Given their experimental susceptibility and this clinical case report, mycobacteriosis should be considered as a differential in guinea pigs exhibiting weight loss in the absence of other clinical signs. With the potential for zoonotic transmission in immunosuppressed individuals, precautions should be taken to safeguard human health in cases of guinea pigs with suspected *M. genavense* infection.

## Background

Mycobacteria are aerobic, non-sporulating bacteria found in many environmental conditions, and these bacteria infect various species, including humans [1–3]. Rodents and rabbits can be essential reservoirs of transmission of mycobacteria between wildlife, domestic animals, and humans [4]. Guinea pigs are used experimentally as a human model for *M. tuberculosis*; however, natural mycobacteriosis has yet to be reported in this species. The following report details the first documented case of *Mycobacterium genavense* infection in a guinea pig and follow-up investigation of two conspecific guinea pigs and two close-contact domestic pigeons.

## Case presentation

A 1.5-year-old female guinea pig (case 1), *Cavia porcellus*, housed in a zoo was found deceased with no premonitory illness. This individual was part of a public education program, was acquired one year prior from a local rescue organization and lived with two clinically healthy female conspecifics.

**Fig. 1:**
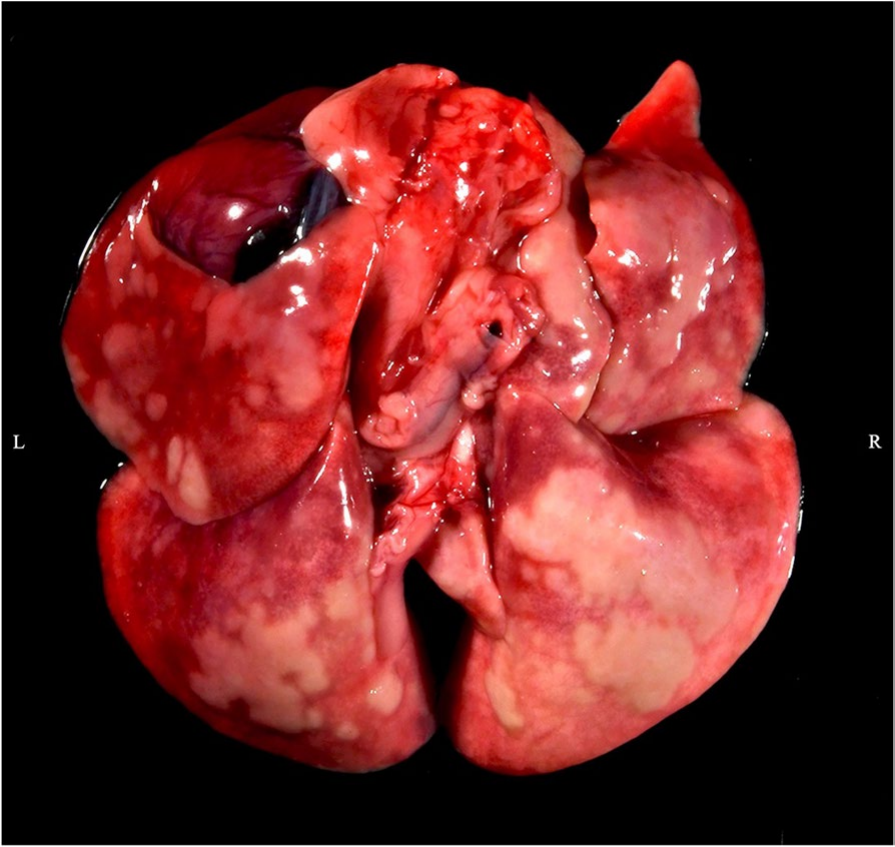
Gross image of post-mortem examination of guinea pig #1. Multifocal to coalescing, raised, firm, pale tan nodules with discrete, irregular margins were noted throughout all lung lobes during the post-mortem examination of guinea pig #1

**Fig. 2:**
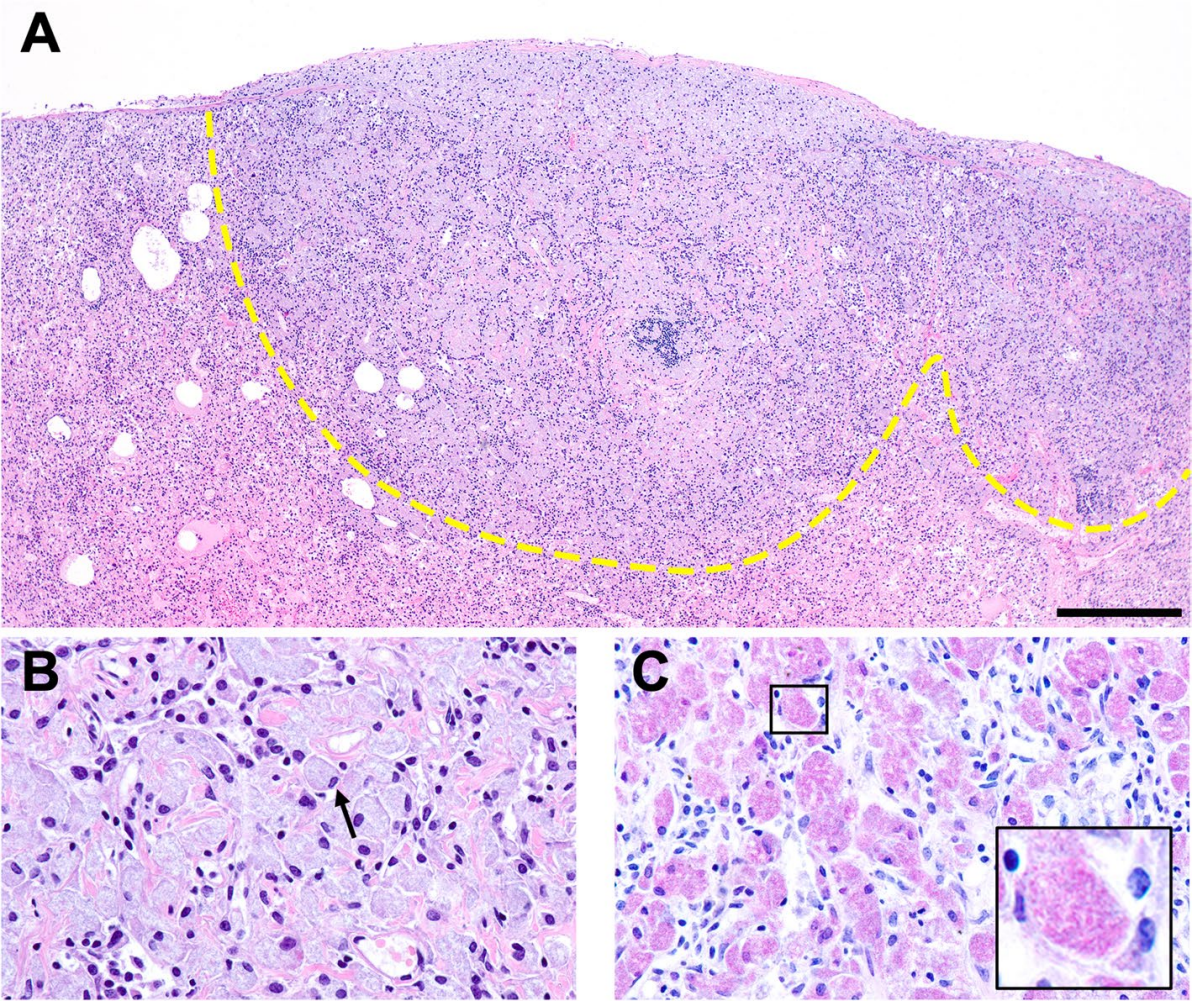
Histologic image from post-mortem examination of case #1. **A** Pulmonary parenchyma and pleura are infiltrated and expanded by inflammatory nodules consisting of macrophages, multinucleated giant cells, lymphocytes, and plasma cells. The dashed line delineates the transition between healthy pulmonary parenchyma and a pulmonary lesion at the top of the image. Hematoxylin and eosin (H&E) stain. 4 × magnification. Scale bar represents 300 microns. **B** Intralesional macrophages (arrow) contain basophilic, rod-shaped bacteria. H&E stain. 40 × magnification. **C** Macrophages (insert) laden with acid-fast staining bacteria. Fite's acid-fast stain. 40 × magnification. Histology sections were examined using a conventional light microscope (Olympus BX51) equipped with a digital camera (Olympus DP26) and cellSens Standard image analysis software (Olympus, Center Valley, PA 18034, USA). Images were acquired at 300 dpi resolution and were edited for scale bar and white balance in Photoshop 23.1.1 (Adobe, San Jose, CA 95110, USA)

Despite a normal appetite, case 1 lost 60 g of body weight (4.6%) over three weeks prior to death based on medical records. On gross examination, over the surfaces of all lung lobes and extending into the parenchyma multifocal to coalescing, raised, firm, pale tan nodules with discrete, irregular margins were observed (Fig. 1). Histopathology revealed nodules composed of clustered foamy macrophages containing numerous bacterial rods. Similar bacteria-laden macrophages were noted within sections of the liver, lungs, heart, palpebral conjunctiva, duodenum, and cecum. The bacterial rods stained strongly with Giemsa, periodic acid-Schiff, and Fite's acid-fast stains, whereas, weakly stained with Ziehl–Neelsen acid-fast stain (Fig. 2). Histology sections were examined using a conventional light microscope (Olympus BX51) equipped with a digital camera (Olympus DP26) and cellSens Standard image analysis software (Olympus, Center Valley, PA 18,034, USA). The spleen and peripheral lymph nodes were considered normal on gross examination and were not examined histologically. Due to high suspicion for mycobacteriosis, a nested polymerase chain reaction (PCR) was performed, first using primers Mb246 and MbR247 with the product from the first amplification used as a template for the second round with primers Mb1 and MbR7 [5]. The product was sequenced using the nested amplification primers. The 16S rRNA gene sequence was 100% identical to 14 strains of *M. genavense*, including the type strain 2289 identified using a BLAST search of the GenBank database. The next closest match was with *Mycobacterium florentinum* at 98.7%. Because *M. genavense* is considered an opportunistic zoonotic pathogen, educational programs with the guinea pigs were ceased, and personal protective equipment (gloves and masks) was required for animal care staff working with the remaining guinea pigs [1].

**Fig. 3:**
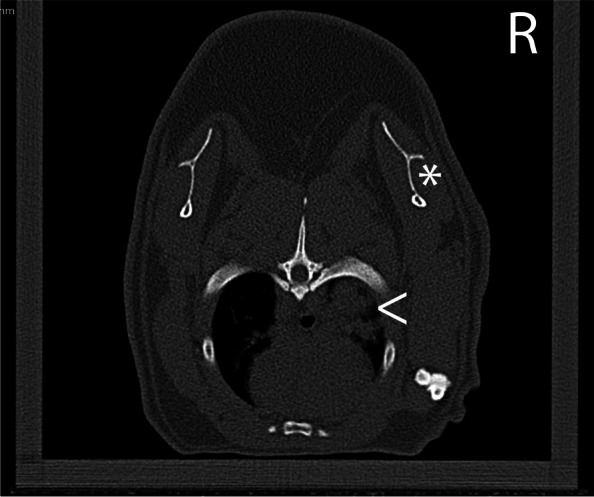
Computed tomography image from case #2. Transverse image at the level of the mid-scapula (*). Within the dorsal aspect of the right cranial lung lobe there are areas of pulmonary consolidation with relatively well-defined margins. ( <) The patient is contained in a fitted box. Image displayed C:320 W2800. Slice thickness 0.69 mm, 120 kV, 139 mA. The pulmonary changes could represent mycobacteriosis as the pulmonary parenchyma was positive on PCR. The distribution of the consolidation, the timeframe of event, and the evolution of the consolidation on the repeat CT would not be considered usual for either aspiration pneumonia nor pulmonary atelectasis

Physical exams and whole-body computed tomography scans were performed on the two conspecific guinea pigs to screen for pulmonary granulomas. Physical examinations were unremarkable. Computed tomography (CT) scans were acquired at a slice thickness of 1 mm, with 139 mA and 120 kV. The raw data were reconstructed in a soft and sharp kernel. Case 2 was anesthetized using isoflurane (Fluriso, MWI Animal Health, Boise ID 83,705 USA) via facemask for induction and maintenance, and the CT scan revealed suspected atelectasis of the entirety of the pulmonary parenchyma within the right hemithorax. To reduce interference with pulmonary atelectasis, CT scans were repeated with the patient awake in a plexiglass chamber (30 cm × 11 cm × 11 cm) approximately one and three hours following the first scan. Both subsequent scans of case 2 revealed focal areas of well-defined, irregular, consolidation of the right pulmonary parenchyma, in the dorsal aspect of the right cranial and right middle lung lobes (Fig. 3). There was no evidence or clinical signs of regurgitation noted. The CT scan of case 3 was performed awake in the same chamber to avoid atelectasis, and the pulmonary parenchyma was within normal limits.

Due to the history of mycobacterial infection in a conspecific and persistent changes within the pulmonary parenchyma identified via cross-sectional imaging, humane euthanasia was elected for case 2. It was re-anesthetized with isoflurane, blood was collected via cardiocentesis, and she was euthanized via intracardiac pentobarbital, and submitted for complete post-mortem examination and PCR of lung tissue for mycobacteria. Based on published reference intervals, a complete blood count (CBC) and chemistry panel were unremarkable [6]. Lung consolidation was noted on gross post-mortem examination. Histopathology revealed evidence of aspiration of gastric contents as well as pulmonary lymphoid hyperplasia (potentially a response to inflammatory disease). Lung tissue was submitted for *Mycobacterium* PCR, which was positive for *Mycobacterium goodii* (99.75%), a strain 93.87% homologous to *Mycobacterium genavense*.

Due to concern for early *M. genavense* infection, potential of zoonosis, and contagious spread to other public education animals, the third conspecific, case 3, was humanely euthanized in the same manner as case 2. A CBC and chemistry panel were performed, and results were unremarkable. Gross post-mortem examination was unremarkable. Lung tissue was submitted for *Mycobacterium* PCR and was negative.

*Mycobacterium genavense* has previously been reported in numerous avian species and is considered one of the most frequent etiologic agents of avian mycobacteriosis in pet birds [7–13]. Because the guinea pigs in this report were previously housed underneath two domestic pigeons, there was suspicion that the pigeons were a potential source of *M. genavense*. Pooled fecal material from the pigeons was submitted for PCR sequencing, and the result was negative. Because two guinea pigs had positive lung PCR results and the sensitivity of detecting mycobacterium in the stool samples was low, the two pigeons were euthanized for post-mortem examination and testing for evidence of *M. genavense*. There was no gross or histologic evidence of mycobacterial infection in either pigeon on postmortem examination. Initial PCR on the pigeon lung tissue revealed *Actinomyces spp.* in one and *Mycobacterium gordonae* in the other. Repeat PCRs were performed due to the possibility of contaminants causing false positive results, which were negative. It was concluded that these pigeons were likely not the source of mycobacteriosis in the guinea pigs. It is possible, however, that case 1 may have been harboring the infection upon arrival to the zoo as pre-shipment diagnostics such as bloodwork and imaging were not performed prior to arrival the previous year, and standard pre-shipment tests likely would not have identified a mycobacterial infection.

## Discussion and conclusions

This is the first documented case of natural *Mycobacterium genavense* infection in a guinea pig. *M. genavense* infection has been reported in numerous small mammal species, including chinchillas, ferrets, rabbits, and squirrels, and avian species including doves, budgerigars, zebra finches, African penguins, and canaries, among others [7–11, 14–18].

Similarities between this and previously reported cases of *Mycobacterium genavense* infection in ferrets and a chinchilla include conjunctivitis, weight loss, and poor body condition [14, 15, 19]. In addition, infection with *M. tuberculosis* has been associated with weight loss in experimentally infected guinea pigs [20]. No organisms were detected via Ziehl–Neelsen acid-fast staining in case 2, which is similar to a ferret diagnosed with *M. genavense* [19]. While case 2 was positive on PCR, it also had evidence of aspiration and pulmonary inflammation; it is unknown if the positive *M. goodii* result was a false positive due to contamination, or evidence of true infection, since it was closely related to *M. genavense*. *M. goodii* is considered an emerging pathogen, commonly associated with nosocomial infections in humans [21, 22]. In animal species, *M. goodii* has been reported in canine patients, a leopard, as well as a spotted hyena with pyogranulomatous pneumonia [23–26].

Prior to presentation, the animals in this report were allowed to be in close contact with the public as educational ambassadors. For human health reasons, strict and thorough diagnostic procedures of close-contact animals, including full post-mortem examinations were performed, as false negative results from other diagnostics (i.e. skin testing, imaging, tracheal washes) would not be acceptable in this situation. In cases of non-tuberculosis mycobacterial pulmonary disease in humans, diagnosis depends on compatible clinical and radiographic findings along with positive sputum samples or positive culture results from bronchial lavage [27, 28]. Unfortunately, tracheobronchial lavage is not considered a routine procedure in guinea pigs due to their small size and laryngeal anatomy, limiting our diagnostic capabilities to non-invasive diagnostics such as CT [29]. While other non-invasive diagnostics, such as tuberculin skin testing, have been reported in guinea pigs for *Mycobacterium tuberculosis* in laboratory settings, the diagnostic sensitivity of this test is unreliable [30]. Further, while bacterial culture was not performed in these cases, culture of mycobacterial organisms could be considered as described in previous reports of *M. genavense* infections in animals [9, 31].

It was concluded that the pigeons in this report were likely not the source of mycobacteriosis in the guinea pigs. It is possible, however, that case 1 may have been harboring the infection upon arrival to the zoo as pre-shipment diagnostics such as bloodwork and imaging were not performed prior to arrival the previous year, and standard pre-shipment tests likely would not have identified an active mycobacterial infection. Future pre-shipment diagnostics to be considered include testing for anti-mycobacterial antibodies to evaluate for potential previous exposure to mycobacterial pathogens.

This is the first documented case of *Mycobacterium genavense* in a guinea pig, with a follow-up investigation unable to determine the source of infection. Mycobacteria can be found in the environment and incidentally in subclinical animals but can also cause severe disease and be difficult to diagnose antemortem. These characteristics made identifying the source of infection challenging. Mycobacteriosis should be included as a differential in guinea pigs presenting with weight loss despite lack of other clinical signs, especially with their high susceptibility to *M. tuberculosis* in experimental settings. Finally, given the potential for zoonotic transmission, precautions should be taken to safeguard health of immunocompromised humans in cases of suspected infection with *M. genavense*.

## Data Availability

The datasets generated and/or analyzed during the current study are available in the GenBank repository, accession number OM760044.

## Abbreviations

CBC: Complete blood count
CT: Computed tomography
H&E: Hematoxylin and eosin
PCR: Polymerase chain reaction
